# TS3 Asn347 corticosteroid-binding globulin (CBG) glycoform deficiency is associated with mortality in human septic shock

**DOI:** 10.1210/jendso/bvag058

**Published:** 2026-03-13

**Authors:** Jessica H Lee, Zeynep Sumer-Bayraktar, R Louise Rushworth, Anastasia Chernykh, Marni Nenke, Morten Thaysen-Andersen, Emily J Meyer, David J Torpy

**Affiliations:** Department of Medicine, University of Adelaide, Adelaide, SA 5000, Australia; Endocrine and Metabolic Unit, Royal Adelaide Hospital, Adelaide, SA 5000, Australia; Endocrine and Diabetes Services, The Queen Elizabeth Hospital, Adelaide, SA 5011, Australia; School of Natural Sciences, Macquarie University, Sydney, NSW 2113, Australia; School of Medicine, The University of Notre Dame, Sydney, NSW 2010, Australia; School of Natural Sciences, Macquarie University, Sydney, NSW 2113, Australia; Department of Medicine, University of Adelaide, Adelaide, SA 5000, Australia; Endocrine and Metabolic Unit, Royal Adelaide Hospital, Adelaide, SA 5000, Australia; Endocrine and Diabetes Services, The Queen Elizabeth Hospital, Adelaide, SA 5011, Australia; School of Natural Sciences, Macquarie University, Sydney, NSW 2113, Australia; Institute for Glyco-Core Research, Nagoya University, Nagoya 464-8601, Japan; Department of Medicine, University of Adelaide, Adelaide, SA 5000, Australia; Endocrine and Metabolic Unit, Royal Adelaide Hospital, Adelaide, SA 5000, Australia; Endocrine and Diabetes Services, The Queen Elizabeth Hospital, Adelaide, SA 5011, Australia; Department of Medicine, University of Adelaide, Adelaide, SA 5000, Australia; Endocrine and Metabolic Unit, Royal Adelaide Hospital, Adelaide, SA 5000, Australia

**Keywords:** corticosteroid-binding globulin, CBG, septic shock, glycosylation

## Abstract

**Context:**

Corticosteroid-binding globulin (CBG) deficiency is an independent predictor of intensive care unit (ICU) mortality in septic shock. CBG glycosylation at the Asn347 site affects neutrophil elastase cleavage susceptibility, altering CBG:cortisol binding affinity, and may underlie this association.

**Objective:**

This work aimed to analyze CBG Asn347 glycoforms in patients with septic shock and determine their relationship to mortality and illness severity.

**Methods:**

CBG Asn347 site glycosylation profiling was performed by mass spectrometry in 128 septic shock patients from a tertiary hospital ICU at day 1 and last day of admission up to 7 days. ICU and 28-day mortality were analyzed by a Cox proportional hazards model, sequentially correcting for illness severity (APACHE score) and total CBG (all glycoforms).

**Results:**

Only triantennary trisialylated (TS3) Asn347 CBG glycoform concentrations were lower in ICU septic shock nonsurvivors than in survivors (29.74 vs 45.16 nmol/L; *P* = 0.007). On Cox multivariate analysis correcting for APACHE score, lowest tertile of TS3 Asn347 CBG glycoform concentration (<27.9 nmol/L) was associated with increased ICU mortality (adjusted hazard ratio [HR] 2.7; 95% CI, 1.2-6.2; *P* = .023) and was the only Asn347 glycoform demonstrating such an association. TS3 Asn347 CBG glycoform concentration less than 27.9 nmol/L was also associated with increased 28-day mortality (adjusted HR 2.2; 95% CI, 1.1-4.4; *P* = .023), which was not the case with lowest tertile of total CBG concentration (<200 nmol/L).

**Conclusion:**

Deficiency of the Asn347 TS3 CBG glycoform is associated with ICU and 28-day mortality in septic shock.

Sepsis is defined as a life-threatening dysregulated systemic inflammatory response to infection, with progression to septic shock characterized by refractory hypotension, metabolic abnormalities, and mulitorgan dysfunction [1]. In-hospital mortality rates of 26% from sepsis [2] and 40% to 60% from septic shock are reported [3-5]. Notably, there have been no major disease-modifying therapeutic advances in recent decades and current treatment remains largely supportive, centered on timely antimicrobial therapy, fluid resuscitation, and catecholaminergic vasoactive agents, with mechanical ventilation and renal replacement therapy (RRT) implemented as clinically indicated.

Our prospective observational study of 135 intensive care unit (ICU) septic shock patients, which provided the samples for this extension study, demonstrated that serum CBG concentrations—comprising all glycoform subtypes—in the lowest tertile (<200 nmol/L; normal reference range, 269-641 nmol/L) were associated with increased ICU mortality (hazard ratio [HR] = 2.0; 95% CI, 0.9-6.0; *P* = .04) and represented the only potentially reversible factor among 4 independent predictors of mortality [6]. Neither hydrocortisone (HC) use nor serum cortisol was associated with mortality [6], consistent with randomized clinical trials of intravenous glucocorticoid administration, which have shown only marginal mortality benefit [2, 3, 7-10] despite cortisol's known immunomodulatory and pressor effects [11, 12]. As with humans, CBG-deficient mice show increased mortality in lipopolysaccharide-induced sepsis models [13]; notably, CBG replacement improved survival compared to that of wild-type controls [14].

Corticosteroid-binding globulin (CBG) is a 383–amino acid, 50- to 60-kDa glycoprotein that binds cortisol in a 1:1 molar ratio with high affinity (K_a_ 76 × 10^6^ L/mol) [15]. In health, 80% of plasma cortisol is bound to CBG, 10% to 15% to albumin, and 5% is free (unbound) [15]. CBG molecular characteristics allow for optimal spatiotemporal distribution of free cortisol [16]; acidosis and pyrexia both reversibly reduce CBG:cortisol binding affinity in vitro, while neutrophil elastase (NE) cleavage of CBG at the reactive center loop (RCL) in vitro irreversibly reduces CBG:cortisol binding affinity by 90% [17, 18], which overall increases free cortisol concentrations at sites of inflammation [17].

CBG is heavily glycosylated with 6 utilized *N*-glycosylation sites [19]. The Asn347 glycosylation site, which lies within the RCL, is only 3 amino acids away from the NE cleavage site at Val344-Thr345. In vitro, glycosylation at the Asn347 site protects against loss of cortisol-binding capacity when incubated with NE [20]; furthermore, volume-enhancing features of the Asn347 glycan protected against NE cleavage of CBG RCL, whereas *N*-acetylneuraminic acid (NeuAc) type sialylation augmented NE cleavage [21, 22].

We hypothesized that specific CBG Asn347 glycoforms concentrations, by virtue of their in vitro effect on CBG cleavage [20-22], may relate to the previously demonstrated mortality effect of total CBG, which encompassed all glycoforms.

## Materials and methods

### Study participants

Samples were taken from our prospective observational study of 135 septic shock patients [6]. Participants were age 18 years or older, admitted to the Royal Adelaide Hospital ICU and receiving intravenous norepinephrine. Septic shock was defined as the presence of a clinically or microbiologically documented infection; 2 or more points on the Sequential [sepsis-related] Organ Failure Assessment (SOFA) score; and norepinephrine requirement for at least 6 hours to maintain a mean arterial pressure greater than 65 mm Hg [1]. Exclusion criteria included expected death within 24 hours; receipt of norepinephrine for more than 48 hours; pregnancy, known conditions that alter cortisol levels including systemic glucocorticoid use for 3 months or longer, hypothalamic-pituitary-adrenal axis disorders, or systemic glucocorticoid administration within the ICU for non–septic shock indications. No study participant received etomidate.

Serum for CBG Asn347 glycosylation profiling had been stored at −80 °C. Samples had been collected on day 1 and the last day of ICU admission (defined as the day of ICU discharge due to recovery or death, or day 7 of ICU admission, whichever occurred first). Samples from 128 patients were analyzed, as 7 patients' samples had insufficient serum. Demographic and clinical outcome data including survival, ventilator use, RRT, Sequential Organ Failure Assessment (SOFA) score, routine laboratory data, and CBG and cortisol concentrations from the original study were used [6]. Serum from 8 healthy individuals (5 women, 3 men) comprised control data.

This study was approved by the Central Adelaide Local Health Network Human Research Ethics Committee (protocol No. R20160208 HREC/16/RAH/29). Written informed consent was obtained from participants or their next of kin.

### Corticosteroid-binding globulin purification and glycopeptide preparation

Mouse 12G2 monoclonal antibody (mAb) (RRID: AB_2632404), produced and purified in-house from 12G2 hybridoma cell culture, was used for CBG immunoprecipitation from serum. 12G2 mAb was coupled to M-280 Tosyl-activated Dynabeads (ThermoFisher Scientific) via 22-hour incubation at 38 °C in 0.1-M borate buffer with 3-M ammonium sulfate. A total of 1-mg tosyl-activated magnetic beads covalently bound to 20 μg of 12G2 mAb were incubated with 125 μL of serum, CBG was then eluted from the beads using 0.1-M glycine, followed by neutralization with 500-mM ammonium bicarbonate, and dried by vacuum centrifugation.

CBG was reconstituted in 8-M urea, followed by reduction and alkylation using 5-mM dithiothreitol (DTT) and 20-mM iodoacetamide (IAA), respectively. This was transferred to S-Trap mini spin columns (ProtiFi) with the addition of phosphoric acid and wash/binding buffer, then centrifuged to trap proteins, and washed 3 times with wash buffer. Trapped proteins underwent overnight trypsin digestion, and the resulting glyco-/peptides were eluted using triethylammonium bicarbonate (TEAB), formic acid, and acetonitrile. The eluted fractions were combined and vacuum dried.

### Mass spectrometric assessment of Asn347 site glycosylation

An aliquot of each glyco-/peptide sample was reconstituted in 0.1% formic acid and loaded on to a trap column custom packed with ReproSil-Pur C18-AQ 5-μm resin (Dr Maisch, GmbH) operated by UltiMate 3000 RSLCnano HPLC system (ThermoFisher Scientific). The injected peptides were separated at a constant flow rate of 300 nL/min at 45 °C on Reprosil-Pur 120 C18-AQ analytical LC column (Dr Maisch GmbH) in reverse phase mode. The mobile phases consisted of 99.9% acetonitrile in 0.1% formic acid (B) and aqueous 0.1% formic acid (A). The nanoLC was connected to a Q-Exactive HF-X Hybrid Quadrupole-Orbitrap mass spectrometer (ThermoFisher Scientific) in positive ion polarity mode. Full MS1 scans were acquired with an automatic gain control (AGC) target of 3 × 10^6^ ions and a maximum fill time of 60 ms, at high resolution of 120 000 full width at half maximum (FWHM) with an m/z range of 450 to 2000.

For Asn347 site glycosylation profiling, tandem mass spectrometry (MS/MS) in parallel reaction monitoring mode was performed with an inclusion list containing m/z values of RCL Asn347 glycopeptide precursor ions based on prior data dependent acquisition discovery. Select glycopeptide ions (z ≥ + 2) were fragmented via higher-energy collision dissociation fragmentation with a normalized collision energy of 30%. Fragment ions were measured at resolution of 30 000 FWHM at m/z 200, with an AGC target of 2 × 10^5^ ions and maximum injection time of 50 ms with a precursor isolation window of m/z 1.4 and a loop count of 10.

For Asn347 glycosylation site occupancy, aliquots of glyco-/peptides were de–*N*-glycosylated via PNGase F (10 U in 20 μL 50 mM ammonium bicarbonate) digestion for 16 hours at 37 °C, followed by liquid chromatography–MS/MS analysis in data-dependent acquisition mode; MS/MS data were collected on the 20 most abundant precursor ions in each MS1 full scan using higher-energy collision dissociation fragmentation with a normalized collision energy of 28%. Fragment ions were measured at resolution of 15 000 (FWHM m/z 200) with an AGC target of 2 × 10^5^ ions and a maximum injection time of 28 ms using a precursor isolation window of m/z 1.2. The data were browsed using Xcalibur v2.2 (ThermoFisher Scientific), and searched against human CBG (UniProtKB, P08185). As deglycosylation by PNGase F deamidates asparagine (N) to aspartic acid (D) residues, it was possible to distinguish between nonglycosylated and formerly glycosylated RCL peptides. Asn347 site occupancy was calculated as a percentage of deglycosylated RCL peptide over total RCL peptide using area under the curve measurements. Relative abundance of each Asn347 glycoforms was obtained using area under the curve measurements. Absolute concentrations of nonglycosylated and deglycosylated RCL peptides, and each Asn347 glycoform, were calculated using a preexisting total CBG concentration measurement via the 12G2 immunoassay for each corresponding sample, obtained previously [6].

For the 8 healthy individuals, the total CBG concentration measurements using 12G2 immunoassay were not available; for the discussion section, the mean total CBG value from previously published data of 73 healthy individuals (459 ± 11 nmol/L) [23] was used to calculate inferred CBG glycoform concentrations.

### Statistical analysis

Pearson correlation was used to determine the relationship between continuous variables. *T* tests were used to compare distributions of continuous variables between categories, using the Welch correction, where appropriate. Cox models were constructed to assess the relationship between the glycoforms and both ICU and 28-day mortality after adjusting for total CBG and day 1 APACHE score (a composite ICU mortality risk score, incorporating age; vital signs; pH; electrolytes; renal, hematologic, hepatic, and neurologic function; as well as preexisting immunocompromise or organ failure). Statistical significance was classified as *P* less than .05. Analyses were undertaken using GraphPad Prism, version 10.0.0 (GraphPad Software). All continuous variables are expressed as mean ± SE unless otherwise stated.

## Results

### Patient characteristics

The study population comprised 128 patients derived from the initial 135 patients in the original study [6]. Baseline demographic and clinical data are summarized in Table 1. These did not differ significantly between those with total CBG of greater than or less than 200 nmol/L as previously reported [6].

**Table 1:** Baseline and clinical characteristics of the study population

Baseline characteristics (n = 128)	
Age, y	63.3 ± 14.3
Sex (% male)	49/128 (38.3%)
BMI	30.4 ± 9.9
Diabetes	43/128 (33.6%)
APACHE II	22 ± 7.0
SOFA	10 ± 3.2
WCC (cells × 10^−9^/L)	18.0 ± 13.3
Albumin, g/L	24 ± 6.2
Lactate, mmol/L	3.75 ± 2.6

Clinical characteristics/data (n = 128)	
HC use (%)	52/128 (40.6%)
RRT (%)	36/128 (28.1%)
No. of days on RRT	0.96 ± 1.8
Norad total dose, μg	54771.5 ± 61353.9
Norad dose/d, μg	16582.5 ± 10616.4
Mechanical ventilation	73/128 (57.0%)
Days in ICU	8.9 ± 8.7
ICU mortality	24/128 (18.8%)

Data are shown as mean ± SD, or n/total n (%).

Abbreviations: BMI, body mass index; HC, hydrocortisone; ICU, intensive care unit; RRT, renal replacement therapy; SOFA, Sequential (sepsis-related) Organ Failure Assessment; WCC, white cell count.

### Corticosteroid-binding globulin Asn347 site glycosylation profile

On day 1 of ICU admission for septic shock, mean site occupancy (% glycosylated) at Asn347 was 76.6 ± 0.6% (range, 57.0%-95.9%), as previously reported [24]. There was a negative association between Asn347 site occupancy and total CBG concentration; however, this did not reach statistical significance (Pearson coefficient *r* = −0.169; *P* = .053).

**Figure 1:**
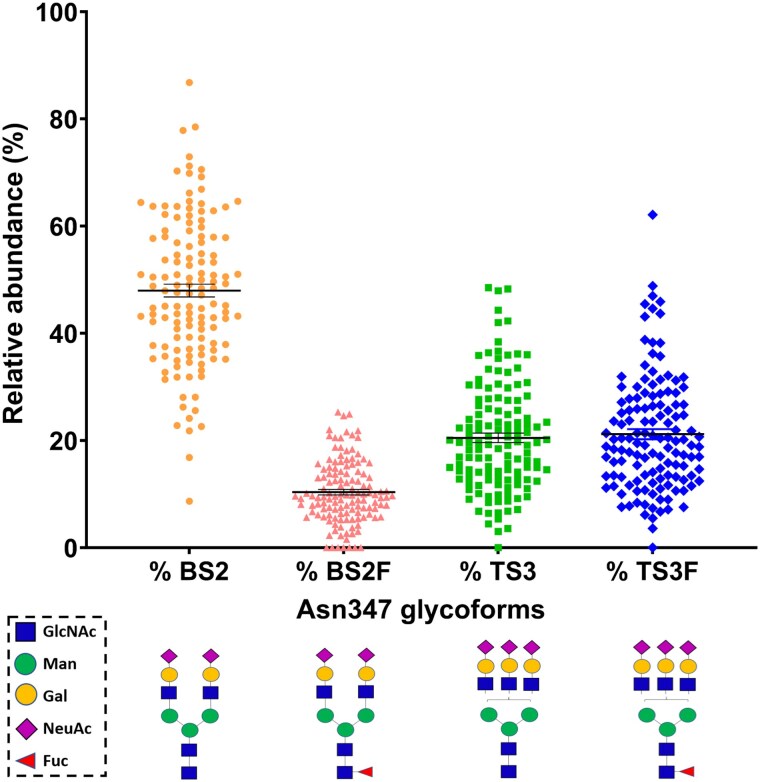
Relative abundance of corticosteroid-binding globulin (CBG) Asn347 site glycoforms in day 1 (intensive care unit admission) septic shock patients. Biantennary disialylated (BS2), biantennary disialylated core-fucosylated (BS2F), triantennary trisialylated (TS3), and triantennary trisialylated core-fucosylated (TS3F) glycans were consistently detected across all samples with mean relative abundance of 48.0%, 10.4%, 20.5%, and 21.1%, respectively. Mean ± SEM shown.

Four types of glycans were consistently found on Asn347 site glycosylation profiling; biantennary disialylated (BS2), biantennary disialylated core-fucosylated (BS2F), triantennary trisialylated (TS3) and triantennary trisialylated core-fucosylated (TS3F) glycans, with a mean relative abundance of 48.0 ± 1.2%, 10.4 ± 0.5%, 20.5 ± 0.9%, and 21.2 ± 0.9%, respectively (Fig. 1), as previously reported [24]. There was no sex difference in absolute glycoform concentrations.

In the healthy sample (n = 8), the mean site occupancy was 69.7 ± 3.1% (range, 29.4%-79.8%) and the relative abundance of Asn347 glycoforms was 49.9 ± 3.1%, 11.0 ± 0.9%, 27.8 ± 3.1%, and 11.4 ± 1.7% for BS2, BS2F, TS3, and TS3F, respectively.

### Day 1 corticosteroid-binding globulin Asn347 glycoforms and septic shock mortality

**Figure 2:**
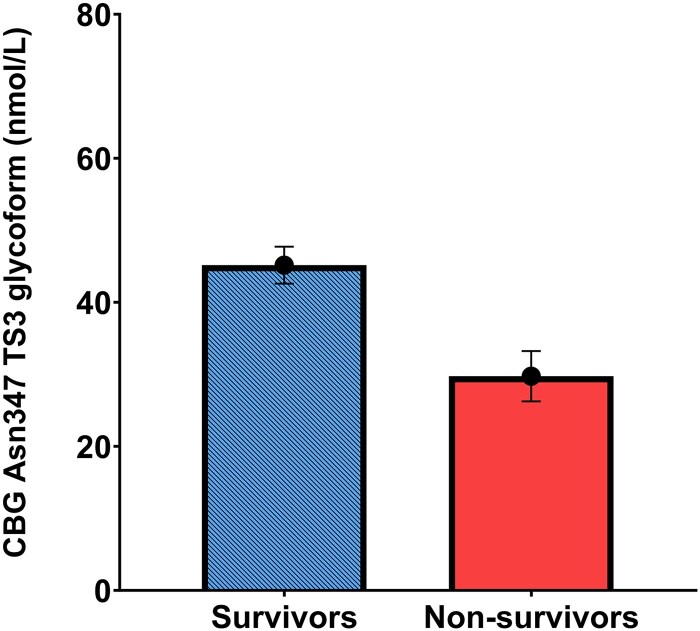
Concentration of corticosteroid-binding globulin (CBG) with triantennary trisialylated (TS3) glycoform at Asn347 site by intensive care unit survival status in septic shock patients. Septic shock survivors 45.16 nmol/L vs septic shock nonsurvivors mean 29.74 nmol/L (*P* < 0.001). Mean ± SEM shown.

CBG Asn347 TS3 glycoform concentrations were lower in septic shock ICU nonsurvivors than in survivors (mean 29.7 ± 3.5 vs 45.2 ± 2.5 nmol/L; *P* < .001) (Fig. 2). The other 3 CBG Asn347 glycoforms were not associated with ICU survival: BS2 (96.9 ± 10.5 vs 103.0 ± 4.7 nmol/L; *P* = .580); BS2F (20.9 ± 3.4 *vs* 22.8 ± 1.6 nmol/L; *P* = .612); TS3F (43.3 ± 7.2 vs 44.4 ± 2.5 nmol/L; *P* = .859). There was no correlation between any of the 4 CBG Asn347 glycoform concentrations and APACHE score or duration of ICU admission.

The relationship between each glycoform and ICU and 28-day mortality was explored using individual Cox regression models, which included day 1 total CBG concentration and APACHE score to control for disease severity. Only models evaluating TS3 Asn347 glycoforms were statistically significant.

**Figure 3:**
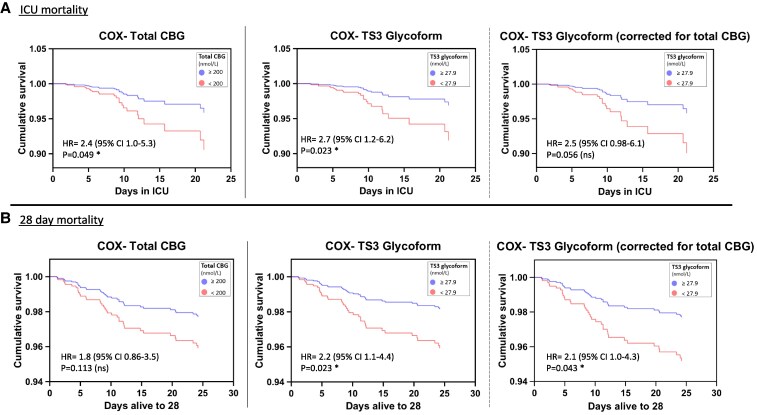
Cox regression analysis for A, intensive care unit (ICU) mortality and B, 28-day mortality for lowest tertiles of total corticosteroid-binding globulin (CBG) (<200 nmol/L) and triantennary trisialylated (TS3) Asn347 CBG glycoform (<27.9 nmol/L), correcting for APACHE score ± additional correction for total CBG in case of TS3 CBG glycoform. Hazard ratio (HR), 95% CI, and *P* value shown inside each graph.

ICU mortality was significantly associated with total CBG concentration (adjusted for APACHE score), with the lowest tertile of total CBG concentration (<200 nmol/L) having an adjusted hazard ratio (HR) of 2.4 (95% CI, 1.0-5.3; *P* = .049). Similarly, the lowest tertile of TS3 Asn347 CBG glycoform concentration (cut point 27.9 nmol/L) adjusted for APACHE score, was associated with increased ICU mortality (adjusted HR 2.7; 95% CI, 1.2-6.2; *P* = .023). When adjusted for both total CBG and APACHE score, the adjusted HR for the lowest tertile of TS3 Asn347 CBG glycoform concentration was 2.5 (95% CI, 0.98-6.1; *P* = .056) (Fig 3).

The 28-day mortality after adjustment for APACHE score was not significantly associated with total CBG concentration tertile (adjusted HR 1.8; 95% CI, 0.86-3.5; *P* = .113). By comparison, the lowest tertile of TS3 Asn347 CBG glycoform concentration was associated with an increased risk of 28-day mortality (adjusted HR 2.2; 95% CI, 1.1-4.4; *P* = .023), and this association remained statistically significant when further adjusted for total CBG concentration, (adjusted HR 2.1; 95% CI, 1.0-4.3; *P* = .043) (see Fig 3).

### Day 1 corticosteroid-binding globulin Asn347 glycoforms and intensive care unit morbidity

There was no correlation between any of the day 1 CBG Asn347 glycoform concentrations and the requirement for or duration of RRT, or duration or dose of inotropic support. Lower CBG Asn347 TS3F glycoform concentrations were associated with a requirement for mechanical ventilation (37.9 ± 2.6 vs 52.6 ± 4.2 nmol/L; *P* = .002) and greater sepsis illness severity, as measured by day 1 total SOFA score (*r* = −0.335; *P* < .001). There was no association between day 1 total SOFA score and any of the other Asn347 glycoforms: BS2 (*r* = 0.033; *P* = .709), BS2F (*r* = −0.100; *P* = .263), or TS3 (*r* = −0.072; *P* = .420).

### Change in concentration of corticosteroid-binding globulin Asn347 glycoforms in septic shock during intensive care unit admission

Glycosylation profiling was also performed on serum from the last day of sampling (by recovery/ICU discharge, death, or day 7, whichever occurred first). Total Asn347 site glycosylation occupancy decreased from day 1 to the last day of ICU admission (76.6 ± 0.6% vs 59.6 ± 1.0%; *P* < .001), both in ICU survivors (76.6 ± 0.7% vs 59.5 ± 1.2%; *P* < .001) and nonsurvivors (76.5 ± 1.4% vs 60.1 ± 2.4%; *P* < .001). There was no difference in Asn347 site occupancy between survivors and nonsurvivors on day 1 (76.6 ± 0.7% vs 76.6 ± 1.4%; *P* = .969) or the last day of ICU admission (59.5 ± 1.2% vs 60.1 ± 2.4%; *P* = .837). The relative abundance of last day of ICU admission BS2, BS2F, TS3, and TS3F Asn347 glycoforms was 51.3 ± 1.2%, 13.1 ± 0.7%, 15.8 ± 0.7%, and 20.3 ± 0.9%, respectively. Mean total CBG concentration from day 1 to last day of ICU admission remained unchanged (276.3 ± 9.1 vs 283.8 ± 10.8 nmol/L; *P* = 0.598).

**Figure 4:**
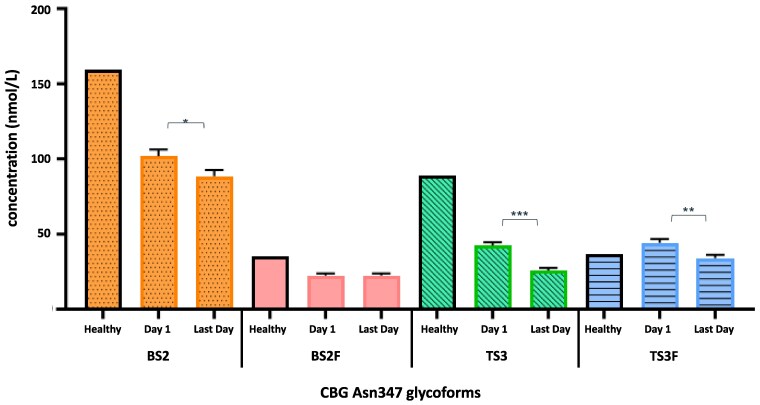
Change in absolute concentration of corticosteroid-binding globulin (CBG) Asn347 glycoforms from day 1 to last day of intensive care unit (ICU) admission. The most pronounced reduction of 38.8% was seen in triantennary trisialylated (TS3), from day 1 to last day of ICU admission in all patients (42.27 vs 25.87 nmol/L; *P* < 0.001), followed by triantennary trisialylated core-fucosylated (TS3F) with 22.9% reduction (44.21 vs 34.08 nmol/L; *P* = 0.002) and biantennary disialylated (BS2) with 13.2% reduction (101.8 vs 88.4 nmol/L; *P* = .027). Concentration of biantennary disialylated core-fucosylated (BS2F) glycoform remained unchanged from day 1 to last day of ICU admission (22.40 vs 22.39 nmol/L; *P* = .993). Inferred concentration of each CBG Asn347 glycoforms in healthy population are also shown [23]. Compared to the healthy physiologic state, by day 1 of ICU admission with septic shock, the relative reduction is estimated to be approximately 36% for BS2 and BS2F glycoforms, 52%, for TS3 glycoform; for TS3F glycoform, there is a relative increase of 22%.

The absolute concentration of TS3 glycoforms were 38.8% lower on the last day of ICU admission compared to day 1 in all patients (42.3 ± 2.2 vs 25.9 ± 1.6 nmol/L; *P* < .001), followed by TS3F with a 22.9% reduction (44.2 ± 2.4 vs 34.1 ± 2.1 nmol/L; *P* = .002) and BS2 with a 13.2% reduction (101.8 ± 4.3 vs 88.4 ± 4.2 nmol/L; *P* = .027). The BS2F glycoform concentration did not change from day 1 to the last day of ICU admission (22.4 ± 1.5 vs 22.4 ± 1.6 nmol/L; *P* = .993) (Fig 4).

Total CBG did not change during ICU admission (day 1 to last day) and there was no effect of HC administration (n = 52), namely, (245.5 ± 12.77 to 253.9 ± 17.64 nmol/L; *P* = .701 [treated]) and (298.3 ± 12.70 to 302.9 ± 13.77 nmol/L; *P* = .080 [untreated]). HC administration did not affect TS3 glycoform reduction, with mean concentration reduction during ICU admission of (−14.6 ± 3.62 [treated] vs −17.4 ± 2.68 nmol/L [untreated]; *P* = .527).

### Last day corticosteroid-binding globulin Asn347 glycoforms and septic shock outcomes

Absolute concentration of last day of ICU admission TS3 glycoform did not differ between ICU survivors and nonsurvivors; (27.19 ± 1.75 vs 19.96 ± 3.54 nmol/L; *P* = .079).

Absolute Asn347 TS3 glycoform concentration on the last day of ICU was negatively associated with last-day total SOFA score (*r* = −0.181; *P* = .047), last day SOFA cardiovascular score (*r* = −0.277; *P* = .002), and last day SOFA central nervous system score (*r* = −0.190; *P* = .037). Absolute concentration of TS3F Asn347 glycoform on the last day of ICU admission showed a negative correlation with last day total SOFA score (*r* = −0.209; *P* = .022). No clinical correlation was observed with BS2 or BS2F glycoforms and SOFA scores.

Lower TS3 Asn347 glycoform concentrations on the last day of ICU were associated with higher total noradrenaline dose administered within the ICU (*r* = −0.197; *P* = .030); there was no such association with other Asn347 glycoforms. Patients requiring mechanical ventilation had lower concentrations of TS3 (22.9 ± 2.1 vs 29.8 ± 2.4 nmol/L; *P* = .030) and TS3F Asn347 glycoforms (29.4 ± 2.4 vs 40.4 ± 3.7 nmol/L; *P* = .014) on the last day of ICU admission. There was no difference in concentrations of BS2 and BS2F glycoforms based on ventilatory requirement.

## Discussion

Asn347 site glycosylation profiling of CBG in septic shock patients revealed a significantly lower concentration of the TS3 Asn347 glycoform of CBG in ICU nonsurvivors; such an association was not seen with the other 3 Asn347 glycoforms. Cox multivariate analyses correcting for APACHE score showed TS3 glycoform concentrations (<27.9 nmol/L) were associated with an HR of 2.7 (95% CI, 1.2-6.2; *P* = .023) for ICU mortality, and 2.2 (95% CI, 1.1-4.4; *P* = .023) for 28-day mortality. These HRs contrast with that of total CBG at 2.4 (95% CI, 1.0-5.3; *P* = .049) for ICU mortality and 1.8 (95% CI, 0.86-3.5; *P* = .113) for 28-day mortality. Even with correction for total CBG concentration, which partly negates the effect of TS3 glycoform (as TS3 concentration is calculated as the relative abundance of TS3 multiplied by total CBG concentration), the increase in ICU mortality and 28-day mortality risk for TS3 remained statistically significant. This suggests that the previously reported association between CBG and mortality in the same septic shock patient population [6], may be largely influenced by the TS3 Asn347 glycoform of CBG. Notably, TS3F, closely related to TS3 and differing only by a single core–located fucose residue, was also associated with reduced mechanical ventilation requirement and lower sepsis severity. While exploratory, this observation raises the possibility that subtle differences in CBG glycoform structure at Asn347 may have functionally relevant effects on critical illness.

The mechanistic explanation for the association between TS3 Asn347 glycoform deficiency and increased septic shock mortality is unclear. It may relate to NE cleavage susceptibility; NE cleavage of CBG RCL at a site only 3 amino acids from the Asn347 glycosylation site irreversibly reduces cortisol affinity by 90% [18]. In vitro, Asn347 glycosylation reduces susceptibility to NE cleavage [20], particularly with volume-enhancing features such as *N*-acetylglucosamine (GlcNAc) branching and core-fucosylation [21, 22]. MS quantification of CBG NE cleavage in septic shock patient serum in a parallel study revealed CBG cleavage is increased in septic shock, and Asn347 glycosylation negatively correlates with percentage of cleaved CBG. In the peripheral circulation, NE-cleaved CBG comprised less than 1% of total CBG [24]; however, this NE cleavage is likely to be more relevant and pronounced at the tissue and cellular level [25]. CBG RCL is also cleaved by other enzymes such as chymotrypsin and pseudolysin (LasB), and hence Asn347 site glycosylation may affect CBG cleavage by other enzymes [21]. Glycan composition at Asn347 alters RCL conformation in vitro [21, 22] and may have allosteric effects on CBG conformation that could alter cortisol-binding affinity directly, or in response to factors such as pyrexia or acidosis. It is also possible CBG Asn347 site glycosylation may have effects beyond cortisol-binding affinity including CBG stability, putative receptor interactions [26], cortisol-related transgene expression [27], or other functional properties.

At day 1 of admission, mean total CBG concentrations were 276.9 ± 9 nmol/L, a 40% reduction compared to healthy matched population values of 459 ± 11 nmol/L [23]. Depletion of total CBG early in septic illness is well established [28, 29]; several mechanisms may be involved, including proteolysis, extravasation, and reduced synthesis. Inflammatory cytokines reduce *SERPINA6* expression [30, 31], and likely increase CBG catabolism, given the rapid decline within 24 hours of interleukin-6 [32] and tumor necrosis factor-α [33, 34] administration, despite the half-life of iodine-labeled CBG being 4.7 to 6.0 days [35]. The inferred mean concentrations of CBG Asn347 glycoforms in healthy individuals for BS2, BS2F, TS3, and TS3F are 159.7 nmol/L, 35.1 nmol/L, 88.9 nmol/L, and 36.4 nmol/L, respectively; thus, there is a relative reduction of 36% for BS2 and BS2F glycoforms, 52% reduction for TS3 glycoform, with an increase of 22% for TS3F glycoform from the healthy state to day 1 of ICU septic shock admission (see Fig. 4).

While total CBG was lower than the healthy reference range, it did not decrease further during ICU admission; however, there was a reduction in the proportion of Asn347 site glycosylated CBG from 76.6% to 59.6% *(P* < .001) over the course of ICU admission. In particular, TS3 glycoform concentration decreased by 38.8% during this period. The mechanism of differential depletion of Asn347 glycoforms is unknown. Possibilities include preferential hepatic synthesis of less-branched biantennary glycoforms in septic shock and/or hepatic dysfunction, perhaps due to altered substrate and enzyme availability for glycosylation or production of CBG with aberrant glycosylation from alternative tissue origins. Hormonal milieu may alter CBG glycosylation, as exposure to insulin, thyroxine, and estradiol have been shown to affect CBG secretion and glycosylation in HepG2 cells [36]; in view of this, the effect of HC administration was assessed in this study, but did not appear to affect the decline in total CBG nor the TS3 glycoform. It is also plausible that triantennary Asn347 glycoforms are more susceptible to metabolism and clearance, resulting in shorter half-life in the circulation.

These observations may have considerable therapeutic implications. Plasma CBG is better correlated to sepsis severity than either total or free cortisol [24] in humans and rodents [25]. CBG knockout mice exhibit increased mortality after lipopolysaccharide administration to simulate sepsis [13]. Krüppel-like factor 15 *(Klf15)* (transcriptional activator of the CBG gene *[Serpina6]*) knockout mice have a 90% reduction in plasma CBG; notably, this excess mortality was reversed by restoration of CBG via CBG complementary DNA transfection [14], supporting a causal relationship between CBG deficiency and mortality. In the context of findings to date, a future attempt at replacement of CBG in septic shock should include adequate quantities of TS3 Asn347 glycoforms. Our results also provide impetus for future research into the association between CBG glycosylation and the protein structure and function, and more broadly into the mechanism by which CBG is associated with clinical outcomes in septic shock, particularly when neither free nor total cortisol concentrations were associated with outcome.

This study has several limitations. The Cox proportional hazards model has demonstrated the independent mortality effect of the TS3 An347 CBG glycoform, but likely has quantitatively underestimated the effect. Stringent corrections for illness severity (APACHE score), and total CBG, which incorporates TS3 glycoform, did not negate the mortality effect of low TS3 CBG glycoform, in contrast with the other CBG glycoforms, but likely has significantly underestimated the effect size of TS3 deficiency on mortality. Methodologically, MS quantification was relative, with absolute concentrations derived from total CBG measurements obtained using the 12G2 mAb immunoassay. CBG for MS analysis was also purified from serum via immunoprecipitation using 12G2 mAb, and the results would be dependent on immunoprecipitation efficacy and absence of glycoform bias in this process. However, the 12G2 mAb based immunoassay has been shown to be reliable and reproducible in many past studies and the agreement of the reported RCL glycoprofiles with our previous studies on human serum CBG glycosylation [19, 21, 22] adds confidence in the findings. Adequacy and reproducibility of immunoprecipitation has also been validated using electrophoresis, Western blots, and MS in the assay development phase.

### Conclusion

This study has shown that deficiency of the TS3 Asn347 CBG glycoform is associated with septic shock ICU and 28-day mortality, an association not seen with the other 3 Asn347 glycoforms. This observation suggests a functional effect of the TS3 Asn347 glycoform that requires further study.

## Data Availability

Restrictions apply to the availability of some or all data generated or analyzed during this study to preserve patient confidentiality or because they were used under license. The corresponding author will on request detail the restrictions and any conditions under which access to some data may be provided.

## Abbreviations

AGC: automatic gain control
BMI: body mass index
BS2: biantennary disialylated
BS2F: biantennary disialylated core-fucosylated
CBG: corticosteroid-binding globulin
FWHM: full width at half maximum
HC: hydrocortisone
HR: hazard ratio
ICU: intensive care unit
mAb: monoclonal antibody
MS: mass spectrometry
NE: neutrophil elastase
RCL: reactive center loop
RRT: renal replacement therapy
SOFA: Sequential (sepsis-related) Organ Failure Assessment
TS3: triantennary trisialylated
TS3F: triantennary trisialylated core-fucosylated
